# The importance of competitive potential in building the international competitiveness of food industry companies: Evidence from Poland

**DOI:** 10.1371/journal.pone.0312512

**Published:** 2024-10-25

**Authors:** Katarzyna Monika Łukiewska

**Affiliations:** Department of Economy’s Competitiveness, Faculty of Economic Sciences, University of Warmia and Mazury in Olsztyn, Olsztyn, Poland; University of Georgia, UNITED STATES OF AMERICA

## Abstract

The economic and social changes taking place in the environment mean that the conditions in which enterprises operate and compete are changing and demanding. This also applies to food industry enterprises, the economic importance is emphasized by many economists. The literature on this subject lacks research on the current factors influencing the competitiveness of food industry enterprises. Thus, the aim of the study was to adopt a multidimensional assessment of the importance of selected components of competitive potential in building the international competitiveness of food industry enterprises. The following research hypothesis was adopted: the key components of the competitive potential of food industry enterprises are intangible. To achieve the main goal and verify the research hypothesis, the empirical study uses information collected using a CATI survey among representatives of food industry enterprises in Poland. The analysis used descriptive statistics, the Mann‒Whitney test, the Kruskal‒Wallis test and exploratory factor analysis, which are rarely used in research on competitiveness. Research has shown that the most important component of competitiveness is intangible assets. The punctuality of deliveries, product quality, company image and relations with suppliers and recipients play special roles. According to factor analysis, four general factors related to competitive potential were distinguished: internal competences and activities, material resources, marketing and clusters, and the market. The results of the study fill a knowledge gap related to the current competitiveness factors of food companies. On a practical level, identifying competitive potential helps improve the ability to compete, better adapt to the environment and gain a sustainable competitive advantage in international markets.

## Introduction

The food industry, the basic link in the food chain, is an important sector in any economy. It has great importance for economic and environmental development and social welfare [1, 2]. It plays an important role in meeting the basic needs of the population and ensuring national and global food security [3, 4]. For this reason, regardless of the structural changes in the economy, it is and will be a strategic industry. The food industry in Poland also plays an important role in the gross domestic product, shaping the labor market and international exchange [5]. Over the past 30 years, it has undergone significant transformations, becoming an important stimulator of economic growth and exports [6]. The main factor in the development of Poland’s agrifood industry was its accession to the EU. Liberalization of trade relations, as well as modernization and adjustment of production plants to EU standards, the inflow of a stream of subsidies, subsidies and foreign investments contributed to a significant increase in the export of agrifood products to the European market [7]. The inclusion and functioning of the structures of the single European market (SEM) are also associated with the increase in the intensity and scope of competition in the international market and the pressure on the domestic market resulting from introducing products of foreign origin and companies locating their production in Poland. In addition, the economic and social changes taking place in the environment make the conditions in which enterprises operate, including those in the food industry, changeable and demanding. This applies, inter alia, to political and economic crises, the COVID-19 pandemic [8], globalization [9], staff shortages [10] and increasing saturation of the food market [11]. For this reason, a constant challenge for Polish food-producing enterprises is gaining and strengthening their competitive position in domestic and foreign markets.

In the literature on this topic, the subjects of competitiveness and development of the Polish agrifood sector are broadly covered. However, the analyses conducted mainly concern production results [e.g., 5, 12], results in foreign trade [e.g., 13–19] or price and efficiency advantages [e.g., 20–24]. There is little research on the current competitiveness factors of the industry and its companies. The considerations presented in the literature on the subject of the conditions for the development of enterprises are often theoretical and refer to other industries or all enterprises without considering the sector of operation [e.g., 25, 26]. Moreover, as Jankowska [27] noted, solutions in one industry are not necessarily successful in another, the specificity of which, e.g., product specificity, is different.

The specificity of the food industry results, among other things, from applying the standards and legal requirements concerning the production, labeling and introduction of products to the market. Various quality management systems are used to ensure that products are safe for consumers and meet specific quality standards. Through these activities, consumers can be confident that their health is protected and they will not be exposed to the harmful effects of consuming products unsuitable for consumption. The issue of food product quality is also considered in the context of the positive and negative features of food, e.g., produced in conventional and organic systems [28].

The problem of insufficient knowledge of international competitiveness factors of food industry enterprises was the main motivation for taking up the topic and seeking to reduce the knowledge gap. In practical terms, awareness of the key competitiveness factors may contribute to improving the ability to compete, better adapting to the requirements of the environment and increasing the international competitiveness of individual food-producing enterprises and the entire food industry. In the context of the above considerations, the aim of this study was to perform a multidimensional assessment of the importance of selected components of competitive potential in building the international competitiveness of food industry enterprises. Consequently, the following research questions (RQs) have been formulated:

RQ1: Which components of competitive potential are assessed as the most important by representatives of food industry enterprises?
RQ2: Do perceptions of the importance of competitive factors differ among food producers and beverage producers?
RQ3: Does the age of the company affect the perception of the importance of the components of competitive potential?
RQ4: Is it possible to isolate a few general factors of competitive potential that determine the international competitiveness of food industry enterprises?

The empirical layer of the study uses information collected in the 2022 primary survey. Opinions on the components of competitive potential were analyzed via statistical methods in the fields of descriptive statistics, the Kruskal‒Wallis test and factor analysis. The use of multivariate methods, to which factor analysis belongs, is extremely useful in the case of economic problems, which are multifaceted, and, thus, their description requires many variables [29]. According to Jurczak and Jurczak [30], factor analysis is a research tool that, owing to its relatively complicated methodology and data volume requirements, is still rarely used. However, the method has great potential as an effective tool to reduce the complexity of observed phenomena. According to the authors, there are no examples of using factor analysis in the field of competitiveness or potential growth of companies, although its results allow for the study of complex problems in the field of competitiveness management with high accuracy and credibility [30]. The method has been used in agrifood research to assess the economic development of rural areas, study the determinants of agricultural income, or determine the determinants of food consumers’ choices, e.g., by Siudek and Vashchyk [31], Sabouri and Solouki [32], Średzińska and Standar [33], Żakowska-Biemans and Gutkowska [34], and Angowski and Bujanowicz-Haraś [35].

The paper is structured as follows. The first section briefly presents the theoretical background of international competitiveness and its influencing factors. The research methods are described in the second section. The next section is devoted to the results of the empirical research. Finally, the conclusions, limitations of the study and suggestions for future analysis are presented.

## The literature review

Competitiveness is one of the main research issues in economic sciences of concern to scientists, politicians and business representatives [36]. The initial interest was related to the attempt to determine the degree of competitiveness of the U.S. and Japanese economies in the face of the intensifying competition between enterprises from these countries [37]. Interest grew due to increasing globalization, which is associated with more open markets and intense changes in market rules at all levels [38]. Competitiveness is a complex and multidimensional category related to various economic theories (including international trade, economic growth and microeconomics), which are ambiguously defined and interpreted in the literature [39–41]. It is often understood as the ability to act and survive in a competitive environment [42] or gaining benefits in a market with increasing competition intensity [43]. Competitiveness concerns entities and their relationships at various levels of economic analysis. An economy’s competitiveness is determined by the competitiveness of its enterprises and industries, and its industrial competitiveness is largely derived from the competitiveness of its members, i.e., enterprises [27, 44]. Notably, the specificity of market conditions means that competition between enterprises takes place in the international market. Even enterprises that do not engage in foreign activities compete with foreign firms in the local and domestic markets.

The international competitiveness of enterprises is shaped by many factors, which can be broadly divided into internal and external factors [45]. Systemic models of enterprise competitiveness emphasize the importance of the competitive potential to achieve specific competitive results. These models included that presented in the 1980s by Buckley et al. [46], which was later referred to and modified by many economists [e.g., 47–49], as well as models in the Polish literature by Gorynia [50], Stankiewicz [51] and Flak and Głód [52]. According to Buckley et al. [46], competitive potential contributes to the competitive process. According to Stankiewicz [51], all tangible and intangible assets are necessary to compete in the market, which, according to Gorynia [50], constitute the main elements of gaining and maintaining a competitive advantage, and, according to Flak and Głod [52], constitute a set of structured resources for implementing the specific functions identical to value chain theory. In various interpretations in the literature, competitive potential is most often equated with the resources, competences and specific skills of a company that contribute to gaining a competitive advantage and achieving competitive results. This approach derives from the resource-based view [53]. According to Gorynia [50], competitive potential may be considered in a narrow and broad sense. From a narrower perspective, all the resources used or possibly used by the enterprise are considered. This applies to primary resources (the entrepreneur’s philosophy and the possibility of gathering know-how), secondary resources (material factors of production, human resources, innovation, distribution channels, and information resources) and results (image, loyalty of the buyer to the product and barriers to switching recipients to other suppliers). From a broader perspective, according to Gorynia [50], competitive potential also includes the culture of the enterprise, its organizational structure, its strategic vision and the behavior appropriate for the enterprise (the process of creating a strategy).

Isolating and classifying individual components of competitive potential is not an easy task, as it requires the use of mutually complementary criteria. The most common method of classification is the division of competitive potential in terms of genera into tangible and intangible resources. As material resources, most authors usually include tangible fixed assets (e.g., land, buildings, equipment, machinery, and means of transport), tangible current assets (e.g., raw materials, materials, and semifinished products) and financial resources (e.g., shares, stocks, and securities) [51]. According to Bednarz [53], intangible resources can be divided into human, market and organizational resources. Human resources are people’s competences, i.e., their knowledge, experience, skills (e.g., creative thinking and problem-solving, entrepreneurial, managerial and leadership abilities) and personality traits (e.g., ability to adapt quickly and creativity). Market resources are relationships with suppliers, buyers, financial institutions, trade unions and other entities, as well as a trade brand, reputation, quality certificates, patents, awards, and the loyalty of buyers. Organizational resources, on the other hand, include technologies, management systems and procedures, culture and organizational structure, as well as interpersonal relations. A similar classification was also presented by Budzyńska-Biernat [54]. A slightly different division of intangible resources was presented by Stankiewicz [51], who distinguished competences that are a feature of the company and employees, intraorganizational relationships and those with entities in the environment, and functional systems, i.e., internally related systems of intellectual activities and activities performed within specific functions, attitudes expressing organizational culture, and opportunities, which are related to accessing and applying specific resources. In turn, Adamik [55] proposed the division of intangible resources into the following four areas: relations, identity and reputation (interactions within and within the environment), organizational culture and attitudes (e.g., the company’s personality and experiences), competences (knowledge and skills) and functional systems (including activities, operations, processes, and activities). There are also other criteria of intangible resources in the literature [e.g., 56, 57].

In the food industry, a relatively large role is played by product quality and quality management, highlighting the need for a holistic and sustainable approach to the food system [58–60]. Product quality can be understood as the degree to which a set of inherent characteristics of an object fulfills requirements [61]. The quality of food products is determined by several important factors. The main parameters include safety, nutritional value, product composition (quality of semiraw materials) and palatability (desirable taste and aroma). In recent years, quality has become an increasingly important decision-making criterion of food consumers, who increasingly seek information about it. Therefore, the quality of a food product can attract consumers to make a purchase, and, according to Alsaleh [62], can even protect the domestic food industry from international competitors.

Studies by previous authors also emphasize the importance of applying quality management principles in building the competitiveness of the food industry [e.g., 58, 63]. For example, the benefits of implementing the ISO 22000 standard include improved efficiency, improved internal procedures, productivity, quality and product safety, anticipating future market trends, improving the company’s image, increasing market share and, as a result, strengthening the company’s competitive advantage [58, 64]. The literature pays special attention to nonmandatory standards, e.g., the ISO 22000:2005 standard, which can provide a solid competitive advantage [58, 65, 66]. Moreover, according to some authors, failure to implement QM initiatives actually prevents effective competition in the domestic and global markets [58, 67].

In the deliberations, there is a noticeable tendency to increase interest in intangible resources as a source of competitiveness and company value. Researchers emphasize the change in the structure of competitive potential and the growing importance of intangible resources and/or indicate that intangible assets determine the durability of competitive advantages more than tangible assets [e.g., 27, 53, 68–71]. The exceptional value of intangible resources results from their uniqueness and difficulties in imitating them. They are specific to each entity. Working them out is a long process; however, over time, they become enriched and increase in value, in contrast to material resources, which are subject to physical and economic deterioration. Moreover, they can be used in various places simultaneously (e.g., in several markets or in several departments of the company) [53, 68, 70]. On the basis of the literature review on the components of competitive potential, the following research hypothesis was formulated:

H1: The key components of the competitive potential of food industry enterprises are intangible.

The many factors of international competitiveness results are due to the complexity of this category. Their importance varies depending on the industry, business profile or market of operation. It is also important to emphasize the existence of critical interplay between factors. This means that individual factors, e.g., the materials and raw materials used, quality, human capital, and a strong brand or company image, can mutually reinforce each other and lead to outcomes such as increased market share, improved financial results and, consequently, increased competitiveness of enterprises and industries.

## Research methods

### Schematic overview of the survey program

**Fig 1:**
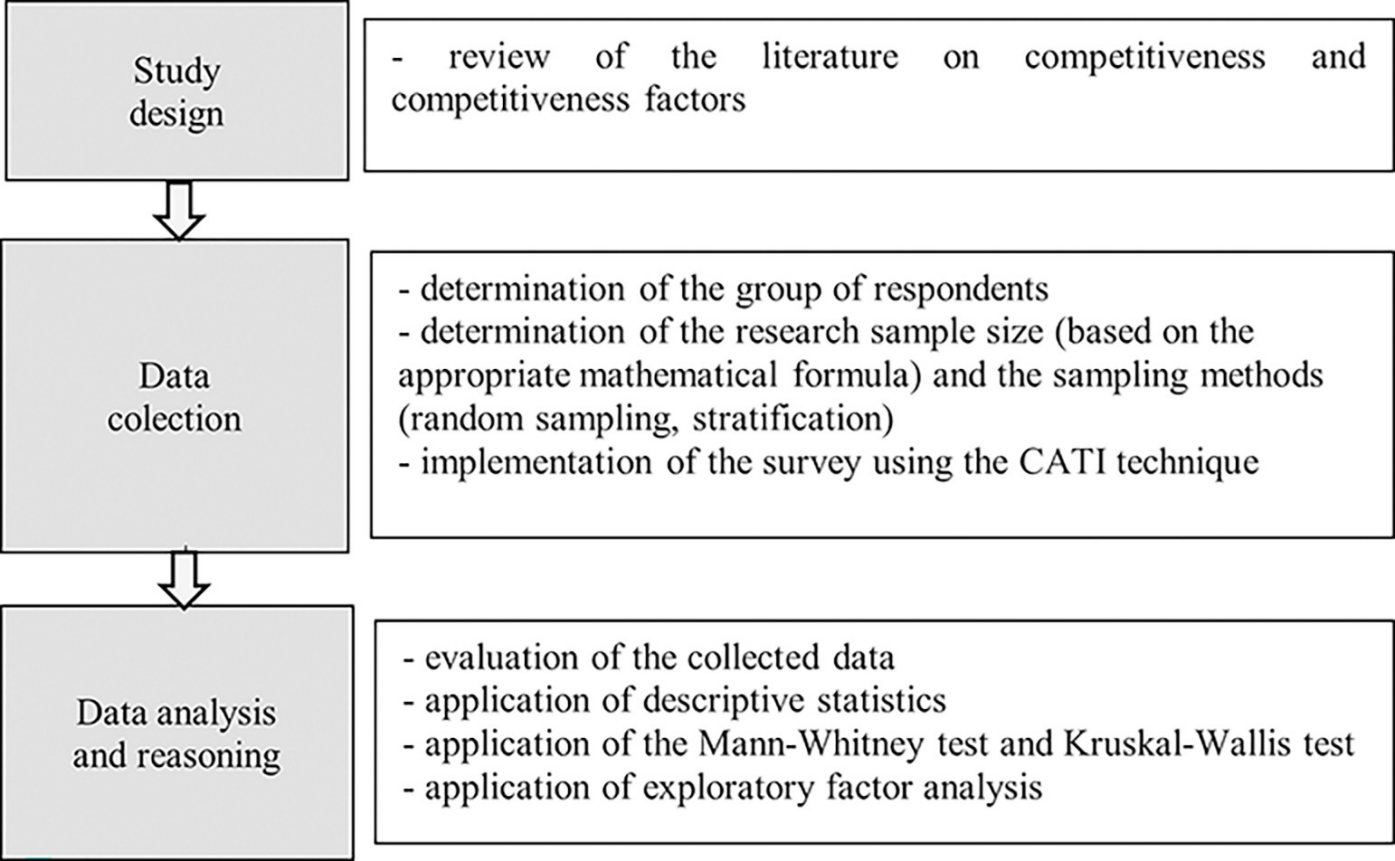
Research process. Source: Own study.

The presented analyses are part of the results of a broader study conducted as part of the project ‘Factors of international competitiveness and development of the Polish food industry’. The study comprised several stages (Fig 1). The first stage involved a systematic analysis of the scientific achievements on international competitiveness and the factors shaping it. In this stage, secondary literature sources were used, i.e., scientific articles and compact publications of national and international scope. The analysis of scientific achievements in this area allowed for the preliminary identification of factors related to the international competitiveness of food enterprises, including competitive potential and the preparation of research tools. The second stage involved carrying out the research. In this stage, the study population was determined, followed by the size of the research sample and the method used for the study. This enabled the survey to be conducted among a defined group of respondents. The third stage of the applied research procedure comprised data analysis, evaluation and presentation of the research results and enabling conclusions. For this purpose, various statistical tools were used, including descriptive statistics, statistical tests and exploratory factor analysis.

### Survey development process

In this study, the research tool used to collect empirical data was a structured interview questionnaire. As previously mentioned, to develop an appropriate tool, an analysis of secondary sources on the competitiveness factors of enterprises and industries was conducted. Several questions were constructed, which were presented in the appropriate order. In the metrics section, the questions concerned the characteristics of enterprises’ business profiles. In the main part, respondents were asked to assess the importance of selected factors in shaping the international competitiveness of food industry enterprises. Tangible and intangible components of competitive potential were considered, as were factors related to innovation, building a food offering in line with current demand and cost‒price factors. A five-point Likert scale [72], which is commonly used to assess attitudes and opinions in social and marketing research [73], was used to assess the importance of individual components.

An important step was determining the group of respondents among whom the interviews were conducted. The study population included entities belonging to Section C of PKD 2007 Industrial Processing, Division 10. Production of food products and Division 11, Production of beverages. To obtain representative data, a stratified random selection of the research sample was used. The criteria for stratification were the PKD department and the size of the enterprise. The schedule was prepared on the basis of the Central Statistical Office data on entities entered in the REGON database. For the study sample to be representative, its appropriate size was also determined. In the course of calculations using the formula for the minimum sample size and assuming a confidence coefficient of 95% and a maximum estimation error of 5% [74], the research sample size was determined at 376 entities (food industry enterprises). The enterprises were located across Poland. The sample structure was dominated by those employing fewer than 10 people (82%). The second group consisted of small enterprises (14%). The smallest group included medium and large enterprises (4%). Notably, such asymmetry in the size of enterprises is consistent with the average size of Polish enterprises in the food industry. An examination of the sample structure according to the duration of operations in the market showed that most had at least twenty years of experience (75%). Almost 16% of the surveyed entities had operated in the market for 10–20 years, and 9% for less than 10 years.

### Survey research implementation

The study was performed using the computer-assisted telephone interviewing technique (CATI). This was based on a predesigned interview questionnaire containing the questions in the correct order. In the CATI technique, the interviewer submits the questions from the interview questionnaire over the telephone, immediately receives the answers and records them in a suitably prepared computer program. This minimizes the risk of errors and ensures the high quality of the method. In addition, using CATI saves costs, guarantees shorter interviews and makes it easier to reach a wide range of respondents. This type of interview is often used in industrial market research, mainly due to entrepreneurs’ time and space indisposition. The survey was conducted in 2022.

### Data assembly and statistical analysis

The result of the conducted quantitative study was raw data, which were subjected to the coding and tabulation. The data were subsequently processed via statistical programs. Descriptive statistics were used to assess the significance of the elements of competitive potential, including the arithmetic mean and the median, lower quartile, upper quartile, minimum and maximum, which are presented in box-plot charts [75, 76]. Factors with higher indicated positional measures are more important [77]. Following the analyses by Çelik and Oral [78] and Renault et al. [79], the corresponding arithmetic mean rating scale was adopted (Table 1).

**Table 1:** Importance of competitiveness components based on the arithmetic mean of scores.

Range of arithmetic mean scores	Importance of competitiveness factors
1.00–1.79	very low
1.80–2.59	low
2.60–3.39	medium
3.40–4.19	high
4.20–5.00	very high

Source: Own study based on Çelik and Oral [78] and Renault et al. [79].

The responses were then analyzed considering the subsector (food producers, beverage producers) and the age (<10, 10–20, >20 years) of the company. The arithmetic mean of the scores in each group was used, and statistical tests were applied to determine if there were statistically significant differences between the groups (separated by subsector and age) in their assessment of competitive factors. In the first case, the nonparametric Mann‒Whitney test was used to test the equality of distribution of the two populations:

$$H_{0}:\;\theta_{1}=\theta_{2}$$ (1)

$$H_{1}:\;\theta_{1}\neq \theta_{2}$$ (2)

The test statistic takes the form:

$$U=n_{1}∙n_{2}+\frac{n_{1}(n_{1}+1)}{2}-R_{1}$$ (3)

*n*_1_, *n*_2_–number of samples,

*R*_1_–sum of ranks awarded to the values of the first attempt.

In the second case, the Kruskal‒Wallis test was used [80, 81]. The test is the nonparametric analog of one-way ANOVA, which can be used when assumptions of normality and/or homoscedasticity are not met [72–74]. The test allows the null hypothesis that the *k* samples are from the same population (with the same medians θ) to be tested:

$$H_{0}:\;\theta_{1}=\theta_{2}=\cdots =\theta_{k}$$ (4)

$$H_{1}:\;not\;all\;\theta_{i}\;are\;equal\;(i=1,2,\ldots ,k)$$ (5)

The test statistics take the form:

$$H=\frac{12}{N(N+1)}\sum_{i=1}^{k}\frac{R_{i}^{2}}{n_{i}}-3(N+1)$$ (6)

$$N=\sum_{i=1}^{k}\;n_{i}$$ (7)

*n*_*i*_−number of observations in a *i* group,

*N*–number of all observations,

*k*–number of compared groups,

*R*_*i*_*−*sum of ranks in a *i* group.

The H statistic has an asymptotic distribution with the degrees of freedom equal to the number of groups k minus 1.

Exploratory factor analysis (EFA) was subsequently used to identify the general factors that influence the international competitiveness of food companies. Factor analysis is a multivariate method in which there are no independent or dependent variables and all variables are rather interdependent. This method identifies the factors essential in determining the essence of the problem under study [82]. An important issue is the transformation of the variables transforming more main variables into a smaller set with minimal loss of information [83]. As Jurczak and Jurczak [84] indicated, the method was proposed by Thurstone [85] on the basis of earlier works by Spearman [86] and Pearson [87].

Factor analysis was preceded by checking the input data and criteria of applicability. Verification of the reliability and internal consistency of the test items was carried out via Cronbach’s alpha [88, 89]:

$$\alpha =\frac{k}{k-1}(1-\frac{\sum_{i=1}^{k}s_{i}^{2}}{s_{sk}^{2}})$$ (8)

*k* –number of items,

*s*_*i*_–variance of the item scores,

*s*_*sk*_–variance of the total scores.

Most authors [e.g., 90–92] indicate that the acceptable value of this indicator should be in the range of 0.70–0.95. Internal consistency checks the correlation within the items in an instrument and shows how well the given items fit a conceptual model [93]. To confirm whether the data from the measurements were sufficient for factor analysis, the Kaiser‒Meyer‒Olkin (KMO) test [94] and Bartlett’s sphericity test [95] were performed. The KMO test statistic is an indicator used to compare the simple and partial correlation coefficients between variables [30]:

$$KMO=\frac{\sum \sum_{i\neq j}r_{ij}^{2}}{\sum \sum_{i\neq j}r_{ij}^{2}+\sum \sum_{i\neq j}q_{ij}^{2}}$$ (9)

$r_{ij}^{2}$–correlation between the variable in question and another,

$q_{ij}^{2}$–partial correlation.

The closer the KMO value is to 1, the stronger the correlation of the index. Typically, when the KMO value is greater than 0.6, factor analysis can be performed [96]. Bartlett’s sphericity test is based on the chi-square statistic, which is sensitive to deviations from the normality of the distribution of results:

$$\chi^{2}=-\left(n-1-\frac{2p+5}{6}\right)\sum_{i=1}^{k}\lambda_{i}$$ (10)

$$df=\frac{1}{2}p\;(p-1)$$ (11)

*p–*number of primary variables,

*n–*cardinality (number of cases),

*λ*_*i*_– *i-th* eigenvalue,

*df–*degrees of freedom.

The null hypothesis of the test assumes that the correlation matrix is an identity matrix, which means there is no correlation between the variables. The rejection of the null hypothesis, in turn, indicates that that the analyzed correlation matrix is significantly different from the identity matrix and that it is reasonable to conduct a factor analysis [30].


$$H_{0}:\;M=I$$ (12)

$$H_{1}:\;M\neq I$$ (13)

*M–*correlation matrix,

*I–*identity matrix.

Then, in accordance with the essence of the factor analysis method, a set of common factors was determined together with the corresponding vector of factor loadings using the principal factor method. The number of common factors was determined on the basis of the following criteria [30]:

1. Kaiser’s rule—The main factors with eigenvalues greater than one are left,
2. Cattell’s criterion—In the crushed stone diagram, the number of eigenvalues after which the graph has a gentle slope indicates the best number of factors.

Among several types of rotation, the most frequently (generally) used varimax method was selected [e.g., 30, 97, 98]. This allows the number of variables with high factor loadings to be minimized through their orthogonal rotation. It was assumed that the relationships of the variables with the factors are strong when the absolute values of its charges are greater than 0.75. Moderate relationships occur when the load values are in the 0.40–0.75 range. Values less than 0.40 should be considered insignificant and omitted [99–101]. The statistical package Statistica 13.3 was used to analyze the results.

## Results and discussion

### Importance of the competitive potential of food industry enterprises

The competitive potential of a company is the foundation of its ability to survive, grow and achieve success in the market. Potential can determine how a company copes with competitive challenges, how effectively it attracts and retains customers, and how quickly it adapts to changing market conditions. Companies with high competitive potential can effectively compete with others, are perceived as more valuable by the customer, gain larger market shares and achieve better financial results. In contrast, companies with weak competitive potential may have difficulty maintaining their position in the market, which, in the long term, may lead to a decrease in their value and profits or even lead to collapse. Therefore, companies that want to be successful in the market must be aware of what elements create their competitive potential and which determine their actual position in the market.

**Fig 2:**
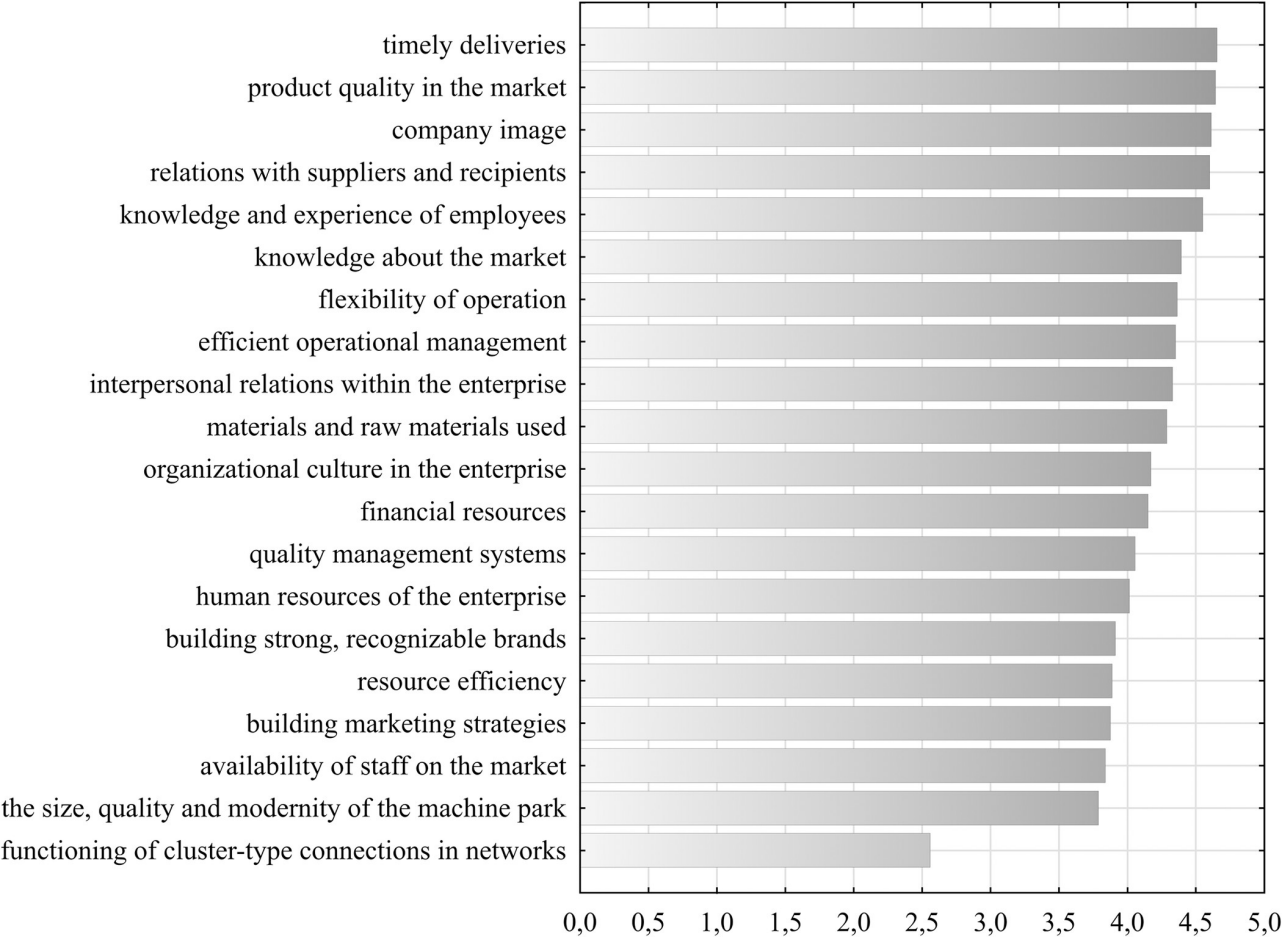
Ranking by arithmetic mean of components of the competitive potential of food industry enterprises in Poland. Source: Own research.

**Fig 3:**
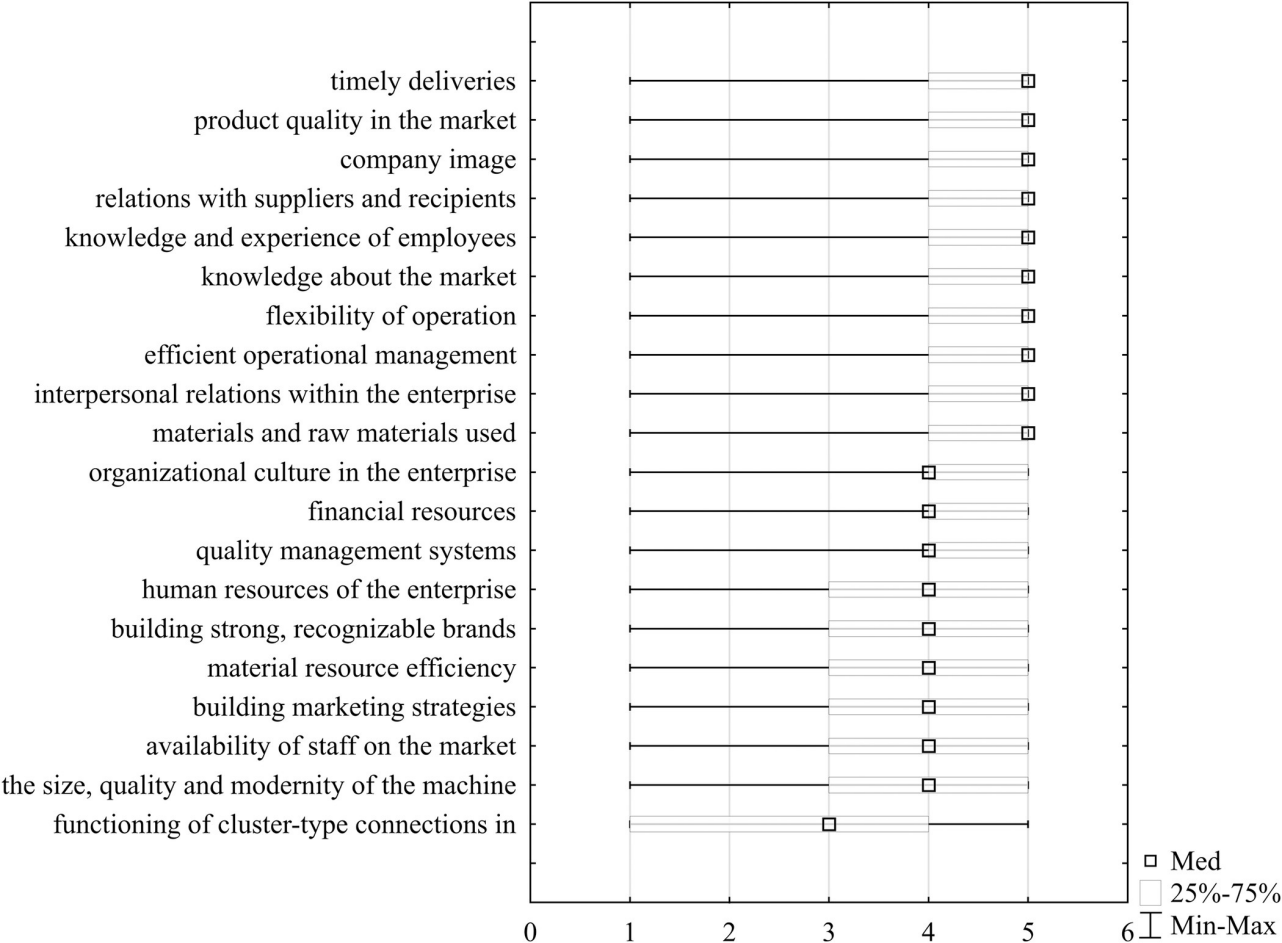
Box plot for components of the competitive potential of food industry enterprises in Poland. Med–median, Min-Max–minimum-maximum. Source: Own research.

In the empirical study, representatives of food industry enterprises were asked for their opinions of the significance of the components of competitive potential selected on the basis of the literature for building an international competitive advantage (on a 5-point Likert scale). The items were ranked by the arithmetic mean (Fig 2), and the basic positional measures of the obtained scores were determined (Table 2 and Fig 3). We then checked whether there were any significant differences in the ratings by subsector and enterprise age (Tables 3 and 4). The survey shows that, in the opinion of the respondents, the most important components of competitive potential are timely deliveries, product quality, company image and relations with suppliers and recipients. The arithmetic mean of the assessments of the indicated components was 4.60–4.65. Notably, these components were characterized by the lowest coefficient of variation, not exceeding 16.5%, indicating high agreement among the respondents’ opinions. According to the respondents, very important elements in building the competitiveness of food enterprises are also the knowledge and experience of employees, knowledge of the market, flexibility of operations, efficient operational management, and interpersonal relations within the enterprise (average 4.33–4.55). The raw materials and materials used are in the next position (average of 4.29). The median of the components indicated was 5, and the upper quartile was 4. This means that 50% of the respondents assigned these elements a score of 5, and 75% of the respondents assigned at least a 4. The differences in the perceptions of these factors among food producers and beverage producers are not statistically significant (based on the Mann‒Whitney test). There were also no significant differences according to the age of the company (based on the Kruskal‒Wallis test).

**Table 2:** Descriptive statistics for assessments of components of the competitive potential of food industry enterprises in Poland.

No	Component	M	Med	D	SD	CV(%)
1	the size, quality and modernity of the machine park	3.79	4	5	1.18	31.12
2	financial resources	4.15	4	5	0.96	23.15
3	human resources of the enterprise	4.01	4	5	1.08	26.96
4	availability of staff on the market	3.84	4	5	1.28	33.43
5	materials and raw materials used	4.29	5	5	0.99	23.14
6	material resource efficiency	3.89	4	5	1.08	27.77
7	product quality in the market	4.64	5	5	0.72	15.50
8	quality management systems	4.06	4	5	1.05	25.90
9	organizational culture in the enterprise	4.17	4	5	0.93	22.33
10	knowledge and experience of employees	4.55	5	5	0.73	16.01
11	interpersonal relations within the enterprise	4.33	5	5	0.85	19.57
12	efficient operational management	4.35	5	5	0.83	19.05
13	knowledge about the market	4.39	5	5	0.80	18.20
14	flexibility of operation	4.36	5	5	0.81	18.67
15	timely deliveries	4.65	5	5	0.69	14.76
16	company image	4.61	5	5	0.68	14.83
17	building marketing strategies	3.88	4	4	1.05	27.02
18	building strong, recognizable brands	3.91	4	5	1.10	28.13
19	relations with suppliers and recipients	4.60	5	5	0.68	14.73
20	functioning of cluster-type connections in networks	2.56	3	1	1.38	53.96

M-arithmetic mean; Med-median; D-dominant; SD-standard deviation; CV-coefficient of variation

Source: Own research.

**Table 3:** Mann‒Whitney test results and arithmetic means by subsector.

Factor		Mann‒Whitney test		Arithmetic mean	
		U	p	F	B
1	timely deliveries	3046.50	0.3302	4.65	4.74
2	product quality in the market	2996.50	0.2636	4.63	4.84
3	company image	3085.00	0.4091	4.61	4.68
4	relations with suppliers and recipients	3323.50	0.8573	4.60	4.68
5	knowledge and experience of employees	3184.00	0.5935	4.54	4.68
6	knowledge about the market	3018.00	0.3657	4.38	4.58
7	flexibility of operation	3383.50	0.9856	4.36	4.37
8	efficient operational management	3036.00	0.3938	4.36	4.21
9	interpersonal relations within the enterprise	3307.50	0.8417	4.33	4.32
10	materials and raw materials used	3274.50	0.7783	4.30	4.11
11	organizational culture in the enterprise	3008.50	0.3712	4.18	4.05
12	financial resources	2767.50	0.1473	4.17	3.89
13	quality management systems	3164.50	0.6015	4.05	4.21
14	human resources of the enterprise	2814.00	0.1853	4.03	3.63
15	building strong, recognizable brands	2252.50	0.0096***	3.88	4.53
16	material resource efficiency	3302.50	0.8409	3.89	3.95
17	building marketing strategies	2770.00	0.1569	3.86	4.21
18	availability of staff ion the market	2826.00	0.1983	3.85	3.53
19	the size, quality and modernity of the machine park	3381.00	0.9820	3.79	3.79
20	functioning of cluster-type connections in networks	2535.00	0.0555*	2.52	3.21

F- food producers, B—beverage producers; ***, ** and * indicate significance at the 0.01, 0.05 and 0.1 levels, respectively.

Source: Own research.

**Table 4:** Kruskal‒Wallis test results and arithmetic means by age of enterprise.

Factor		Kruskal-Wallis test		Age of enterprise (in years)		
		H	p	<10	10–20	>20
1	timely deliveries	0.79	0.6741	4.66	4.76	4.63
2	product quality in the market	3.71	0.1565	4.88	4.69	4.61
3	company image	0.55	0.7595	4.63	4.52	4.63
4	relations with suppliers and recipients	0.48	0.7851	4.56	4.69	4.59
5	knowledge and experience of employees	0.61	0.7382	4.63	4.60	4.53
6	knowledge about the market	0.12	0.9417	4.34	4.47	4.38
7	flexibility of operation	0.41	0.8164	4.25	4.40	4.37
8	efficient operational management	1.71	0.4246	4.34	4.26	4.37
9	interpersonal relations within the enterprise	2.59	0.2736	4.16	4.37	4.34
10	materials and raw materials used	1.39	0.5002	4.06	4.40	4.29
11	organizational culture in the enterprise	0.71	0.7002	4.19	4.13	4.18
12	financial resources	2.86	0.2391	4.00	3.98	4.21
13	quality management systems	0.46	0.7949	4.13	4.11	4.04
14	human resources of the enterprise	5.19	0.0746*	4.00	3.68	4.09
15	building strong, recognizable brands	1.48	0.476	4.16	3.84	3.9
16	material resource efficiency	0.2	0.9043	3.88	3.89	3.89
17	building marketing strategies	1.46	0.4822	4.00	3.79	3.88
18	availability of staff on the market	5.6	0.0607*	3.75	3.50	3.92
19	the size, quality and modernity of the machine park	6.15	0.0462**	3.66	3.52	3.86

***, ** and * indicate significance at the 0.01, 0.05 and 0.1 levels, respectively.

Source: Own research.

The respondents considered company’s organizational culture, financial resources, and quality management systems highly important to building international competitiveness (arithmetic mean 4.06–4.17). The median indicates that 50% of the respondents rated the importance of these components as 4 or 5. According to the respondents, to compete effectively in international markets, food industry companies must also have adequate human resources (average 4.15). The respondents subsequently indicated building strong brands; material resource efficiency; building marketing strategies; the availability of staff in the market; and the size, quality and modernity of the machine park (average 3.79–4.01). According to the positional measures, 50% of the respondents rated these indicators at least 4, and 75% rated them at least 3. The Kruskal‒Wallis test indicated significant differences in the perception of building strong, recognizable brands among food and beverage producers. Beverage producers emphasized the greater importance of this component in building competitiveness. Furthermore, differences were observed in the perceptions of the importance of components such as the human resources of the enterprise; the availability of staff in the market; and the size, quality and modernity of the machine park, depending on the age of the company. These components were considered more important by representatives of enterprises over 20 years old and of young enterprises under 10 years old.

According to the respondents, the functioning of cluster-type connections in networks is of average importance. The arithmetic mean and positional measures of ratings for these components were the lowest among all the analyzed components (the mean was 2.56, median was 3, first quartile was 1, and third quartile was 4). This component was also characterized by the greatest diversification of responses (the coefficient of variation was 53.96%). Notably, beverage producers rated the importance of this factor higher than food producers.

In this study, the highest scores (arithmetic mean 4.33–4.65, median 5) were awarded to nonmaterial components. Thus, the hypothesis that intangible components are the key components of the competitive potential of food industry enterprises has been confirmed. The obtained results are largely consistent with the conclusions of other researchers. The leading role of this type of competitive potential in shaping the competitive advantage of Polish medium and large food industry enterprises was also indicated in the study by Bednarz [53] and in food industry enterprises located in Greater Poland Voivodeship in the study by Budzyńska-Biernat [102]. The key importance of intangible components in the success of enterprises was also emphasized in the study by Jankowska [27] on the construction industry, the study by Lubomska-Kalisz [69] on the competitiveness of small and medium-sized enterprises and the study by Grego-Planer [70] on the so-called mysterious masters, i.e., companies that are in the first, second or third place on the European market, are leaders in the Polish market, have revenues below EUR 3 billion and, at the same time, the public has little awareness of them.

Among the intangible elements, the respondents considered that timely deliveries and relations with suppliers and recipients played important roles. As Michalczyk [103] noted, good relationships between all links in the food chain are extremely important, especially as they are spread across three basic sectors: the food industry, agriculture and trade. According to Dąbrowska et al. [104], the outbreak of the coronavirus pandemic resulted in the need to increase the frequency of contact between companies and suppliers to monitor the situation. Michalczyk [103] noted that, owing to food trends related to the diversification of food and the performance of additional functions by food, e.g., health, in addition to the existing extensive connections, short supply chains are also formed. Owing to greater transparency and direct contacts, they make it possible to adjust the supply faster, better meet consumers’ changing preferences and improve the competitiveness of the entities participating in them. The author confirms that, considering the specificity of the food industry, which is a relatively fast sales market, timeliness, economy and "agility" are important attributes for enterprises, according to Michalczyk [103]. The research carried out by Kamińska [105] shows, in turn, that the timeliness of deliveries and their compliance with orders and a responsible approach to the obligations incurred are the keys to the success of Polish companies in the German market. Research has shown that the most important components of the competitiveness of food industry enterprises also include product quality and company image, which are emphasized relatively often in the literature. Kociszewski and Szwacka [106] indicated that in the food industry, enterprises can gain a clear competitive advantage by adopting a competitive strategy of introducing new products to the market and creating a positive image of a modern company that produces products of unwavering quality. According to the authors, the image associated with high product quality is also of particular importance in the case of highly processed food products characterized by a lower saturation of needs.

Notably, cluster-type connections in networks were definitely the lowest-rated element of competitive potential by the respondents. However, the literature indicates that links between entities belonging to the cluster may be an important component of competitiveness in the food industry [e.g., 107]. According to Klimczuk [106], cooperation between entities may contribute to improving all functional and resource spheres, and is particularly important for the research and development sphere, production, generally invisible resources or supply logistics. An example of an efficiently functioning cluster in the food industry is the "Food Cluster of Southern Wielkopolska" [108]. Kowalski and Weresa [109] focused on the new dimension of competitiveness, which gained special importance after the outbreak of the COVID-19 pandemic, i.e., "relational competitiveness". It is related to the ability to use and create relational capital, i.e., the potential possessed by an organization in connection with its external relations, enabling access to intangible assets, including the knowledge of customers, suppliers, government or industry associations. Therefore, functioning in clusters appears to be an increasingly important future source of competitiveness.

### Building the international competitiveness of the food industry

Building international competitiveness is a strategic, complex and multi-faceted process It should take into account the possibility of adapting to a dynamically changing environment, Factors of international competitiveness largely determine the company’s policy and the formulation of development strategies. Managers should define key areas in their entities that will allow them to build a competitive, targeted advantage. This study identifies areas that affect the international competitiveness of food industry enterprises. For this purpose, an exploratory factor analysis was used.

The factor analysis was carried out in several steps. In the first step, the reliability of the scale was measured. Following the factor analysis procedure, the scale’s reliability was measured in the first step. The obtained Cronbach’s alpha index of 0.872320 is clearly greater than 0.70, as recommended by Hair et al. [95] as an acceptable level of reliability. Then, tests of the applicability of the functional analysis were carried out by KMO and Bartlett (Table 5). The p value for the Bartlett sphericity test was significantly lower than 0.05, confirming that the analyzed dataset is not an identity matrix. However, the KMO value of 0.889 was clearly greater than 0.6. Therefore, it should be considered that both tests confirmed the validity of using factor analysis [110].

**Table 5:** KMO and Bartlett’s tests.

Test	Value
Kaiser–Meyer–Olkin measure of sampling adequacy	0.889
Bartlett’s test of sphericity	
•approximate chi-square	5330.004
•degree of freedom	190
•level of significance (p value)	0.0000

Source: Own research.

**Fig 4:**
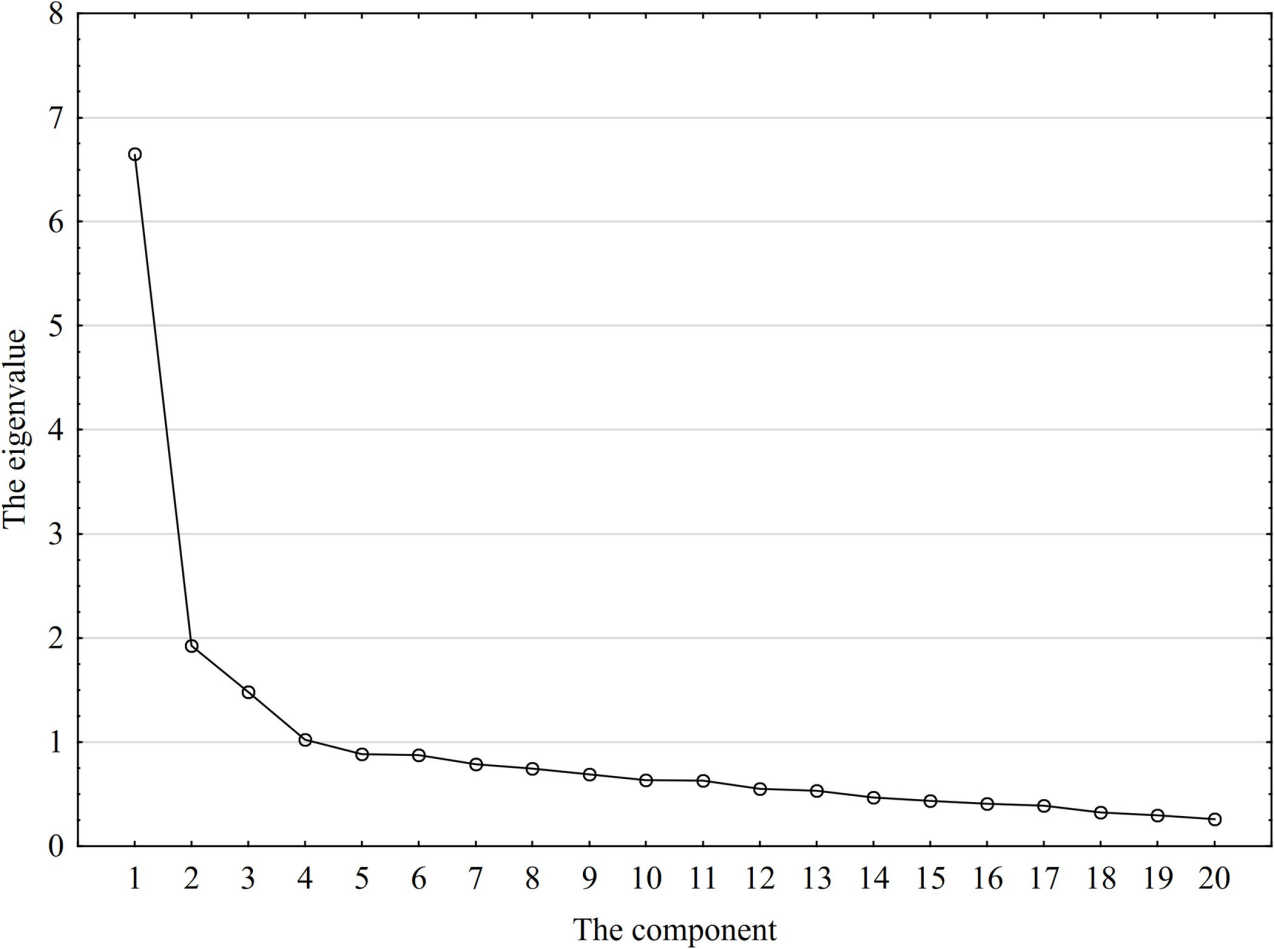
Crushed stone. Source: Own research.

The rest of the study focused on selecting the optimal number of common factors. In accordance with the adopted criteria, the structure of the eigenvalues was assessed on the "scree" chart (Fig 4), and the factors with eigenvalues greater than one were selected (Table 6). On this basis, 4 important factors were adopted. Moreover, with such a selection, each factor explains at least 5% of the total variance and is associated with at least three initial variables. Then, the orthogonal rotation of the system (Varimax) was applied, which allowed us to emphasize their charges and simplify the structure of connections between the initial variables. Notably, in the analyzed case, applying the criterion of a sufficient proportion at the level of 75% as indicated by some authors [e.g., 97, 111] proved of little use. This would require adopting 9 factors that would explain 75.33% of the variance. As Jurczak and Jurczak [84] noted, many factors would cause the model to be too fragmented, and some of them would be related to only one initial variable, contradicting the use of factor analysis. According to Hedhili and Boudabbous [98], the percentage of variance explained must be greater than 50%. Similar proportions were also determined in the works of Krasowska and Banaszuk [101] and Feizabadi and Gorj [83].

**Table 6:** Eigenvalues and variance contribution rates.

Common Factor	Characteristic Root	Variance Contribution Rate (%)	Cumulative Variance Contribution Rate (%)
F_1_	6.652124	33.26062	33.2606
F_2_	1.925627	9.62814	42.8888
F_3_	1.481768	7.40884	50.2976
F_4_	1.024116	5.12058	55.4182

Source: Own research.

**Fig 5:**
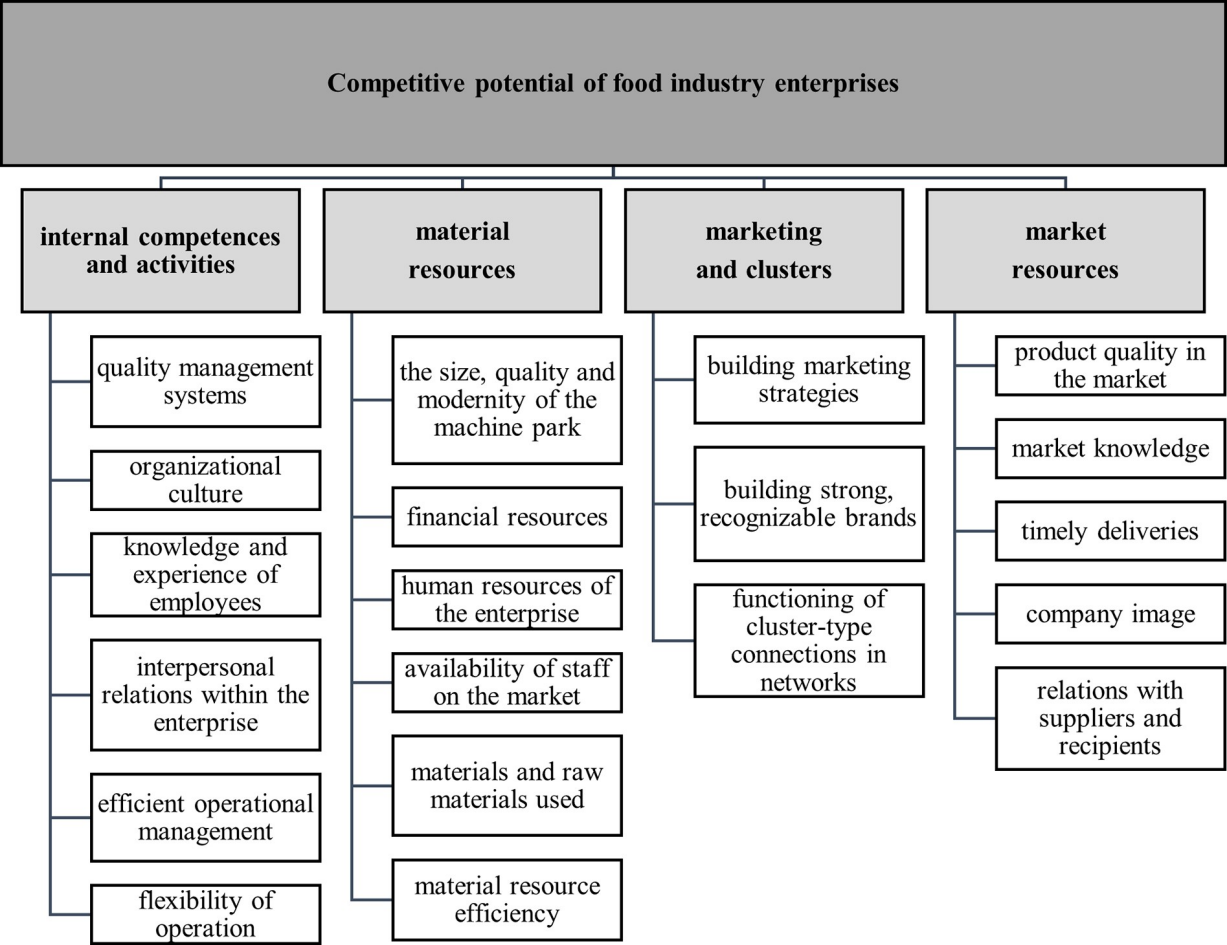
Components and factors of the competitive potential of food industry enterprises in Poland. Source: Own research.

The obtained four factors accounted for 55.42% of the variable resources included in the adopted structure of the variables. The first factor accounted for the greatest amount of variance, i.e., 33.26%. It refers to the selected skills and knowledge of employees and activities undertaken in the enterprise. It was defined as "internal competences and activities" and included the following variables: quality management system, organizational culture in the company, knowledge and experience of employees, interpersonal relations within the company, efficient operational management and flexibility of operations (Table 7 and Fig 5). The second factor explained 9.63% of the variance, concerning the size and effectiveness of the use of material elements in competitive potential. It is characterized by a high value of loads for the following variables: the size, quality and modernity of the machine park; financial resources; the human resources of the company; the availability of staff in the market; the materials and raw materials used; and the material resource efficiency. Owing to the assigned variables, this factor can be described as "material resources". The third factor accounted for 7.41% of the variance and was determined by three variables: building marketing strategies, building strong, recognizable brands, and functioning in networks of connections such as clusters. This has been referred to as "marketing and clusters". The fourth factor accounted for 5.12% of the variance. It consists of the following variables: product quality in the market, market knowledge, timely deliveries, company image, and relations with suppliers and recipients. The fourth factor has been called "market resources".

**Table 7:** Description of the factors influencing the competitive potential of food industry enterprises.

Factor	Variable	Factor loading	Factor name
F_1_	quality management systems	0.697146	internal competences and activities
	organizational culture in the enterprise	0.693371	
	knowledge and experience of employees	0.659471	
	interpersonal relations within the enterprise	0.601915	
	efficient operational management	0.652238	
	flexibility of operations	0.571613	
F_2_	the size, quality and modernity of the machine park	0.520077	material resources
	financial resources	0.683989	
	human resources of the enterprise	0.795545	
	availability of staff in the market	0.711816	
	materials and raw materials used	0.491060	
	material resource efficiency	0.502977	
F_3_	building marketing strategies	0.777212	marketing and clusters
	building strong, recognizable brands	0.763014	
	functioning of cluster-type connections in networks	0.667213	
F_4_	product quality in the market	0.491778	market resources
	knowledge about the market	0.470608	
	timely deliveries	0.756899	
	company image	0.647859	
	relations with suppliers and recipients	0.666949	

Source: Own research.

On the basis of the ratings of the components comprising the individual factors, it was found that market resources (F_4_) and internal competences and activities (F_1_) were by far the most important in building competitiveness (Table 8). The arithmetic mean of the ratings was greater than 4.20 (4.58 and 4.35, respectively), the median was 5, and the first quartile was 4. Material resources (F_2_) were also important. The fourth factor, marketing and clusters (F_4_), played a relatively small role (the lowest mean score—3.45, the lowest median and bottom quartile—3, the lowest dominant—4).

**Table 8:** Descriptive statistics for factors related to the competitive potential of food industry enterprises.

Common Factor	Factor names	M	Med	Q_1_	Q_3_	D
F_1_	internal competences and activities	4.35	5.00	4.00	5.00	5.00
F_2_	material resources	3.99	4.00	3.00	5.00	5.00
F_3_	marketing and clusters	3.45	3.00	3.00	5.00	4.00
F_4_	market resources	4.58	5.00	4.00	5.00	5.00

M-arithmetic mean; Med-median; Q_1_-first quartile; Q_3_-third quartile; D-dominant

Source: Own research.

The conducted research has academic and applied value. The conclusions obtained here have direct implications for managers of food enterprises who formulate competitive strategies. Research has shown that the key to maintaining and improving the competitiveness of food industry enterprises in the international market is to emphasize two functional areas in the enterprise, i.e., market resources and internal competences and activities. Market resources enable not only acquisition but also maintenance of key market segments, as well as adapting to new trends and challenges. The indicated high competences and well-organized internal activities will allow for effective, profitable and flexible competition. The functional areas are intangible, and it seems that in the current economy, they will be more important than traditional material resources. However, the mutual reinforcement of resources from different functional areas will make it possible to optimize the objectives to be achieved and to build greater and more sustainable international competitiveness. The research study concerns both food enterprises operating in foreign and the domestic market, which compete with food importers.

## Conclusion

Enterprises operating in the food sector should consciously shape their competitiveness. To operate in the domestic and foreign markets and develop in the long term, they must increase their competitive potential, giving them an advantage over their competitors. The original empirical research shows that the most important components of competitive potential are intangible elements. Timely deliveries, product quality, the company’s image and relations with suppliers and recipients are of particular importance. According to the respondents, very important elements in building the competitiveness of food companies include the knowledge and experience of employees, knowledge of the market, flexibility of operations, efficient operational management, interpersonal relations within the enterprise, and raw materials and materials used. In addition, there were no significant differences in the assessment of these factors among food and beverage producers or among companies with small, medium and large business experience.

An important added value of the study was determining functional areas that affect the international competitiveness of food industry enterprises using exploratory factor analysis. The following four areas have been identified: internal competences and activities, material resources, marketing and clusters and market resources. According to the respondents, the most important factors were market resources, followed by competences and internal activities.

The presented research has not only academic but also practical value. Defining the direction of building competitive potential may contribute to improving the ability to compete and creating strong and lasting competitive positions of food industry enterprises in domestic and foreign markets. The survey indicated that intangible resources are key to building the international competitiveness of food enterprises. However, it is worth emphasizing that intangible and tangible resources should be treated as complementary, reinforcing each other and creating synergistic effects.

This study has several limitations. The analyses do not consider the broad profile of activity, including the company’s size or directions of product exports. Moreover, the assessment of competitive potential (resources and competences) should be considered as part of the research on the factors of competitiveness of enterprises producing food products and the entire food industry. Subsequent studies will analyze factors related to innovation and the elements of industry 4.0, the market considering current food trends and contemporary economic processes.

## Supporting information

S1 Questionnaire Questionnaire survey. (DOCX)

S1 Data (XLSX)
