# Supplementary material for: The importance of competitive potential in building the international competitiveness of food industry companies: Evidence from Poland
Source: PLoS One. 2024 Oct 25;19(10):e0312512. doi: 10.1371/journal.pone.0312512 (PMC11508086; doi:10.1371/journal.pone.0312512)
Supplement: S1 Questionnaire — (DOCX) [file pone.0312512.s001.docx]

**QUESTIONNAIRE SURVEY**

**Factors of international competitiveness and development of the Polish food industry**

**PART I.**

1. Age of the company:

- up to 2 years
- 2-9 years
- 10-20 years
- over 20 years

2. Size of company:

- small (up to 49 employees)
- medium (50-249 employees)
- large (250 and more employees)

3. Share of export sales:

- up to 10%
- 10-39%
- 40%-60%
- over 60%

4 Company capital:

- exclusively Polish
- exclusively foreign
- mixed with dominant Polish capital
- mixed with dominant foreign capital

5. Main sector of activity:

- 10.1 Processing and preserving of meat and production of meat products
- 10.2 Processing and preserving of fish, crustaceans and molluscs
- 10.3 Processing and preserving of fruit and vegetables
- 10.4 Manufacture of vegetable and animal oils and fats
- 10.5 Manufacture of dairy products
- 10.6 Manufacture of grain mill products, starches and starch products
- 10.7 Manufacture of bakery and farinaceous products
- 10.8 Manufacture of other food products
- 10.9 Manufacture of prepared animal feeds
- 11 Manufacture of beverages

**PART II.**

1. Please specify, the impact of selected economic processes on the operation of food industry companies in Poland?

| No. | Specification | Evaluation  (1 - very low important, 5 - very high important) | | | | |
| --- | --- | --- | --- | --- | --- | --- |
| 1 | Globalisation, causing e.g. dissemination of global brands | 1 | 2 | 3 | 4 | 5 |
| 2 | EU membership | 1 | 2 | 3 | 4 | 5 |
| 3 | Increasing competition | 1 | 2 | 3 | 4 | 5 |
| 4 | Development of information and communication technologies | 1 | 2 | 3 | 4 | 5 |
| 5 | Emphasis on innovation | 1 | 2 | 3 | 4 | 5 |
| 6 | Development of commercial transnational corporations | 1 | 2 | 3 | 4 | 5 |
| 7 | Economic partnership/free trade agreements between the EU and third countries such as Japan (EPA), Canada (CETA) | 1 | 2 | 3 | 4 | 5 |
| 8 | Digitalisation and development of Industry 4.0 | 1 | 2 | 3 | 4 | 5 |
| 9 | Brexit | 1 | 2 | 3 | 4 | 5 |
| 10 | COVID-19 pandemic | 1 | 2 | 3 | 4 | 5 |
| 11 | Sustainability trends | 1 | 2 | 3 | 4 | 5 |

1. Please specify which direction of action will be crucial for building the competitiveness and development of your company (please indicate the 1 most important):

- development of tangible factors (e.g. fixed assets, finances, human resources)
- development of intangible factors (e.g. knowledge, competences, relations, systems, attitudes)
- building a cost-price advantage
- building an offer tailored to the needs of customers
- development of innovation

1. Please assess how important the following tangible and intangible factors are from the perspective of building your company's competitiveness and development?

| No. | Specification | Evaluation  (1 - very low important, 5 - very high important) | | | | |
| --- | --- | --- | --- | --- | --- | --- |
| 1 | the size, quality and modernity of the machine park | 1 | 2 | 3 | 4 | 5 |
| 2 | financial resources | 1 | 2 | 3 | 4 | 5 |
| 3 | human resources of the enterprise | 1 | 2 | 3 | 4 | 5 |
| 4 | availability of staff on the market | 1 | 2 | 3 | 4 | 5 |
| 5 | materials and raw materials used | 1 | 2 | 3 | 4 | 5 |
| 6 | material resource efficiency | 1 | 2 | 3 | 4 | 5 |
| 7 | product quality in the market | 1 | 2 | 3 | 4 | 5 |
| 8 | quality management systems | 1 | 2 | 3 | 4 | 5 |
| 9 | organizational culture in the enterprise | 1 | 2 | 3 | 4 | 5 |
| 10 | knowledge and experience of employees | 1 | 2 | 3 | 4 | 5 |
| 11 | interpersonal relations within the enterprise | 1 | 2 | 3 | 4 | 5 |
| 12 | efficient operational management | 1 | 2 | 3 | 4 | 5 |
| 13 | knowledge about the market | 1 | 2 | 3 | 4 | 5 |
| 14 | flexibility of operation | 1 | 2 | 3 | 4 | 5 |
| 15 | timely deliveries | 1 | 2 | 3 | 4 | 5 |
| 16 | company image | 1 | 2 | 3 | 4 | 5 |
| 17 | building marketing strategies | 1 | 2 | 3 | 4 | 5 |
| 18 | building strong, recognizable brands | 1 | 2 | 3 | 4 | 5 |
| 19 | relations with suppliers and recipients | 1 | 2 | 3 | 4 | 5 |
| 20 | functioning of cluster-type connections in networks | 1 | 2 | 3 | 4 | 5 |

1. Please specify the current state of cost advantages of Polish food producers over EU competitors:

| No. | Specification | 1  much lower | 2  slightly lower | 3  similar | 4 slightly higher | 5  slightly higher | hard to say |
| --- | --- | --- | --- | --- | --- | --- | --- |
| 1 | the cost of agricultural raw materials in Poland | 1 | 2 | 3 | 4 | 5 | 6 |
| 2 | labour costs in Poland | 1 | 2 | 3 | 4 | 5 | 6 |
| 3 | costs of electricity in Poland | 1 | 2 | 3 | 4 | 5 | 6 |
| 4 | costs of production factors in Poland | 1 | 2 | 3 | 4 | 5 | 6 |
| 5 | costs of food distribution in Poland | 1 | 2 | 3 | 4 | 5 | 6 |
| 6 | total production costs in Poland | 1 | 2 | 3 | 4 | 5 | 6 |
| 7 | level of processing margins in Poland | 1 | 2 | 3 | 4 | 5 | 6 |

1. Please specify the current state of price advantages of Polish food products over EU competitors.

Prices of food products produced in Poland in comparison with products from other EU countries are:

- much lower
- little lower
- similar
- bit higher
- much higher
- hard to say

1. Please assess how important the following price and cost factors are from the perspective of building your company's international competitiveness and growth?

| No. | Specification | Evaluation  (1 - very low important, 5 - very high important) | | | | |
| --- | --- | --- | --- | --- | --- | --- |
| 1 | the cost of agricultural raw materials in Poland | 1 | 2 | 3 | 4 | 5 |
| 2 | labour costs in Poland | 1 | 2 | 3 | 4 | 5 |
| 3 | costs of electricity in Poland | 1 | 2 | 3 | 4 | 5 |
| 4 | costs of production factors in Poland | 1 | 2 | 3 | 4 | 5 |
| 5 | costs of food distribution in Poland | 1 | 2 | 3 | 4 | 5 |
| 6 | total production costs in Poland | 1 | 2 | 3 | 4 | 5 |
| 7 | level of processing margins in Poland | 1 | 2 | 3 | 4 | 5 |

1. Please assess how important are the factors related to building the food offer from the perspective of building international competitiveness and development of your company?

| No. | Specification | Evaluation  (1 - very low important, 5 - very high important) | | | | |
| --- | --- | --- | --- | --- | --- | --- |
| 1 | the development of functional foods due to the growing interest in health-oriented foods | 1 | 2 | 3 | 4 | 5 |
| 2 | the development of organic food | 1 | 2 | 3 | 4 | 5 |
| 3 | development of convenience foods (e.g. ready-made flour and potato dishes, frozen meat and fish dishes) | 1 | 2 | 3 | 4 | 5 |
| 4 | development of minimally processed foods | 1 | 2 | 3 | 4 | 5 |
| 5 | development of traditional foods | 1 | 2 | 3 | 4 | 5 |
| 6 | personalisation of food products (e.g. taking into account the needs of seniors, people with increased physical activity) | 1 | 2 | 3 | 4 | 5 |
| 7 | promotion of local products | 1 | 2 | 3 | 4 | 5 |
| 8 | development of foods that reduce calorie intake (so-called 'light foods') | 1 | 2 | 3 | 4 | 5 |
| 9 | development of products associated with fashions for specific diets (e.g. lactose-free, gluten-free) | 1 | 2 | 3 | 4 | 5 |
| 10 | development of products imitating products of animal origin | 1 | 2 | 3 | 4 | 5 |
| 11 | diversity of the range of products on offer | 1 | 2 | 3 | 4 | 5 |
| 12 | development and dissemination of own brands | 1 | 2 | 3 | 4 | 5 |
| 13 | creation of a product brand with unique values for the consumer | 1 | 2 | 3 | 4 | 5 |
| 14 | prosumption, i.e. active involvement of consumers in the process of developing new products on the market | 1 | 2 | 3 | 4 | 5 |
| 15 | speed of response to customer signals | 1 | 2 | 3 | 4 | 5 |

1. Please assess how important the following innovation-related activities are from the perspective of building your company's international competitiveness and development?

| No. | Specification | Evaluation  (1 - very low important, 5 - very high important) | | | | |  |
| --- | --- | --- | --- | --- | --- | --- | --- |
| 1 | development of innovative products | 1 | 2 | 3 | 4 | 5 | |
| 2 | use of innovative raw materials (e.g. spirulina, chia seeds) | 1 | 2 | 3 | 4 | 5 | |
| 3 | implementation of innovative intelligent packaging | 1 | 2 | 3 | 4 | 5 | |
| 4 | implementation of innovative packaging made of renewable or biodegradable raw materials | 1 | 2 | 3 | 4 | 5 | |
| 5 | the use of nanotechnology in packaging development | 1 | 2 | 3 | 4 | 5 | |
| 6 | creating new manufacturing technologies and techniques, including automation of production | 1 | 2 | 3 | 4 | 5 | |
| 7 | use of intelligent robots and machines | 1 | 2 | 3 | 4 | 5 | |
| 8 | computerisation of logistics |  |  |  |  |  | |
| 9 | innovative, modern ways of reaching the customer | 1 | 2 | 3 | 4 | 5 | |
| 10 | innovative, modern management systems | 1 | 2 | 3 | 4 | 5 | |
| 11 | the use of information systems |  |  |  |  |  | |
| 12 | starting cooperation with research centres | 1 | 2 | 3 | 4 | 5 | |
| 13 | implementation of innovative methods of distribution | 1 | 2 | 3 | 4 | 5 | |
| 14 | application of innovative methods in the field of advertising and promotion | 1 | 2 | 3 | 4 | 5 | |
| 15 | application of data mining analysis and evaluation of large databases | 1 | 2 | 3 | 4 | 5 | |
